# Targeting ferroptosis with miR-144-3p to attenuate pancreatic β cells dysfunction via regulating USP22/SIRT1 in type 2 diabetes

**DOI:** 10.1186/s13098-022-00852-7

**Published:** 2022-06-27

**Authors:** Shanshan Zhang, Xiao Liu, Jihong Wang, Fengjuan Yuan, Yali Liu

**Affiliations:** 1 School of Medicine of Pingdingshan University, Middle Section of Chongwen Road, Xincheng District, Pingdingshan, 467000 Henan China; 2 Shool of Nursing, Doctor of Philosophy in Nursing, Philippine Women’s University, Manila, Philippines

**Keywords:** Type 2 diabetes mellitus, Ferroptosis, miR-144-3p, Pancreatic β cells, USP22

## Abstract

**Background:**

Recently, ferroptosis has been implicated in the pathologic process of several diseases including type 2 diabetes mellitus (T2DM). However, molecular mechanisms underlying ferroptosis in T2DM remain obscure.

**Methods:**

Twenty four mice were included in this study. T2DM model mice were established by a high-fat diet combined with streptozotocin injection. INS-1 cells were stimulated with high glucose (HG). Cell viability was detected by CCK-8 kit. The levels of GSH, MDA, iron, and lipid ROS, and SOD activity, were detected by the corresponding kits. The interaction between miR-144-3p and USP22 was validated by dual-luciferase reporter assay. The relationship between USP22 and its substrate was verified using Co-IP and ubiquitination assays. The mRNA and protein expressions were examined by RT-qPCR and western blot, respectively. The functions of β cells in vitro and in vivo were evaluated glucose-stimulated insulin secretion test and HOMA-β, respectively.

**Results:**

Ferroptosis occurred in the pancreas of T2DM mice and HG-induced INS-1 cells. Silencing miR-144-3p blocked the effect of HG on the cell viability and accumulation of lipid peroxides, thereby improving the insulin secretion in INS-1 cells. Mechanistically, USP22 is a direct target of miR-144-3p, which could stabilize SIRT1 expression, thereby suppressing ferroptosis. Overexpressing USP22 attenuated deleterious roles of HG in INS-1 cells; but its roles were reversed by up-regulating miR-144-3p. In vivo study demonstrated that miR-144-3p antagomir exerted an anti-hyperglycemic effect and regulated the ferroptosis-related proteins in the pancreas.

**Conclusion:**

The up-regulation of miR-144-3p suppressed USP22/SIRT1 to induce ferroptosis, which causes pancreatic β cells dysfunction, thereby promoting T2DM development.

**Supplementary Information:**

The online version contains supplementary material available at 10.1186/s13098-022-00852-7.

## Background

Diabetes mellitus, a group of metabolic disorders with abnormalities of insulin secretion, insulin action, or both resulting from various etiologies is defined by elevated serum glucose levels or persistent hyperglycemia [1]. As one of the most widespread and devastating metabolic diseases, diabetes mellitus is the ninth leading cause of death globally [2]. With the economic development and population ageing, China has witnessed one of the most dramatic rises in diabetes mellitus prevalence in the world [2]. A recent study also reported that China exhibited the highest prevalence of diabetes mellitus among 195 countries and territories [3]. In China, the striking rise in diabetes mellitus prevalence is mainly attributed to type 2 diabetes mellitus (T2DM). The prevalence of T2DM was reported to be 382 million in 2013, which was predicted to rise to 592 million in 2035 across 130 countries [4]. T2DM is mainly characterized by chronic hyperglycemia owing to the dysfunction of pancreatic β-cells and insulin resistance. There are multiple factors causing the dysfunction of pancreatic β-cells in T2DM, such as inflammation, aging, and oxidative stress [5].

As a newly discovered iron-dependent cell death, ferroptosis is mainly caused by overwhelming lipid peroxidation, which has been reported to be associated with various metabolic disorders in recent years [6]. Glutathione peroxidase 4 (GPX4) is an antioxidant enzyme of dissipating the accumulation of lipid peroxidate, of which functional activation requires glutathione (GSH) as a cofactor [7]. Cellular GSH synthesis relies on cysteine, which is mostly acquired by cysteine-glutamate transporter (xC^−^) system [7]. Therefore, the repression of GPX4 and/or xC^−^ system directly leads to the activation of ferroptosis. The other characteristic of ferroptosis is a marked increase in cellular irons concentration. Recently, aberrant iron status has been implicated in T2DM in several clinical studies [8–10]. It has been proposed that an excessive iron deposition in the pancreatic islet pancreas leads to the dysfunction of pancreatic β-cells, thereby contributing to T2DM development [11, 12]. Based on these, we supposed that ferroptosis has a pivotal role in pancreatic β-cell dysfunction, which might be a novel therapeutic target of T2DM. Nevertheless, very little is known about the molecular mechanism behind ferroptosis of pancreatic β-cell so far.

As one type of non-coding RNAs, microRNAs (miRNAs) are capable of repressing gene expression to take part in various biological processes [13]; consequently, the abnormal expression of miRNAs is associated with various disease manifestation. In the past few decades, numbers of dysregulated miRNAs have respectively been identified in serum, pancreatic islets, and adipose tissues from patients with T2DM [14]. A growing number of studies documented that some of these dysregulated miRNAs were involved in various processes related to diabetes, including pancreatic β-cells differentiation and insulin secretion [15]. In multiple previous publications, miR-144-3p has been identified to be up-regulated in T2DM [16, 17]. Besides, Karolina et al. [18] demonstrated that miR-144-3p up-regulation contributes to the pathogenesis of T2DM by suppressing insulin signaling. Nevertheless, the effect and mechanism by which miR-144-3p affects the pancreatic β-cell functions in T2DM were largely unknown. A recent study reported that miR-144-3p could enhance hyperglycemia-induced reactive oxygen species (ROS) generation, thereby aggravating diabetic cardiomyopathy [19].

In this study, we sought to investigate whether miR-144-3p impairs the function of pancreatic β-cells via activating ferroptosis in T2DM. Furthermore, we also explore the mechanism underlying the induced-ferroptosis role of miR-144-3p.

## Methods

### Animal and T2DM model establishment

Twenty-four C57BL/6J mice (SPF grade, male, 18–22 g) were purchased from Experimental Animal Center of Zhengzhou University (Zhengzhou, China). Before the start of the experiment, mice were acclimated to a new environment for 1 week. In this study, T2DM model was established by combining a high-fat diet (HFD) which induces insulin resistance and a low dose of streptozotocin (STZ) administration that causes initial β-cell dysfunction. Briefly, mice were randomly assigned into three groups (n = 8/group): control, T2DM model, and T2DM model + miR-144-3p antagomir groups. Mice in the control group were fed with a regular chow diet, whereas other mice were fed with an HFD. After 4 weeks of dietary manipulation, HFD-fed mice received an intraperitoneal administration of STZ (50 mg/kg), while the control group mice were given a 0.1 M citrate buffer. The successful establishment of T2DM model was affirmed by the blood glucose levels of mice (≥ 16.7 mM).

Blood was collected from the caudal vein of mice after a 10 h-fasting to separate serum, and subsequently stored at − 80 °C for examining the serum levels of glucose and insulin. At the end of the experiment, pancreatic tissues were extracted from mice after anesthetization. Half of pancreas tissue samples were immersed in 4% buffered paraformaldehyde overnight for fixation; then the fixed tissues were embedded in paraffin and cut into pieces (4 μm sections). To investigate the histopathological change of the pancreas, the sections were subjected to hematoxylin–eosin (H&E) staining. The remaining pancreas tissue samples were stored at a − 80 °C refrigerator until next analysis. All experiments involving animals were reviewed and approved by the School of Medicine of Pingdingshan University Academy Animal Experimental Ethical Committee (Approval No. LCKY2021-58).

### Cell culture and transfection

832/3 rat insulinoma cell line (INS-1) purchased from Sigma-Aldrich (MO, USA) were grown in DMEM medium containing 10% FBS and antibiotics at 37 °C in a CO_2_ incubator.

Mimic and inhibitor for miR-144-3p (miR-144-3p mimic and miR-144-3p inhibitor) and the corresponding negative controls (NC mimic and NC inhibitor) were supplied by the GenePharma company (Shanghai, China). Gene-specific (USP22 or SIRT1) overexpression plasmids or shRNAs suppling by the GenePharma were transfected into cells to overexpress or knock down the expression level of proteins. According to standard protocols, INS-1 cells were transfected with the stated plasmids using Lipofectamine 2000 (Life Technologies, CA, USA) and harvested at 48 h post transfection for further experiments.

### Biochemical parameters detection

Blood glucose level was determined with a glucose meter. The serum was obtained from blood by centrifugation at 3000 rpm for 10 min. Serum levels of iron and insulin were estimated using Iron Assay Kit (Abcam, MA, USA) and Mouse Insulin ELISA kit (Mercodia, Uppsala, Sweden), respectively. Insulin concentration in cellular supernatant was detected by radioimmunoassay (RIA). Superoxide dismutase (SOD) activity in pancreatic tissues was evaluated using SOD Colorimetric Activity Kit (Life Technologies, CA, USA). The levels of GSH and malondialdehyde (MDA) in pancreatic tissues and INS-1 cells were detected by GSH Assay Kit (Abcam, MA, USA) and Lipid Peroxidation (MDA) Assay Kit (Sigma-Aldrich, MO, USA), respectively. Lipid peroxidation in INS-1 cells was evaluated by lipid ROS levels determined by the BODIPY^™^ 581/591 C11 (Life Technologies, CA, USA) method as Martinez et al. [20] described.

Additionally, the homeostasis model assessment (HOMA) was used to evaluate the function of pancreatic β-cells, HOMA-β = 20 × fasting insulin/(fasting blood glucose-3.5) [21].

### RT-qPCR

Total RNA of tissues or cells was isolated using TRIzol reagent (Life Technologies, CA, USA). Then, RT-qPCR analysis was conducted as described previously [22]. After determining the RNA concentration, first-strand cDNA synthesis was performed using 2 µg RNA with iScript Reverse Transcription Supermix (Bio-Rad, CA, USA), according to the manufacturer’s protocol. RT-qPCR reaction was performed in the ABI 7900 H sequence detection PCR system with SYBR Green PCR Master Mix (Takara, China) kit according to the following reaction conditions: Initial denaturation at 95 °C for 10 min; 40 cycles of 93 °C for 15 s, 58 °C for 60 s. The primer sequences used in this study were as followed: miR-144-3p (forward (F): 5′-GGCCGGCGTACAGTATAGATGA-3′, reverse (R): 5′-GTGCAGGGTCCGAGGT-3′); USP22 (F: 5′-CATGACCCCCCTTTCATGGCCT-3′, R: 5′-GATGTTCTGGTGACGGGTGT-3′); SIRT1 (F: 5′-TCATTCTGACTGTGATGACGA-3′, R: 5′-CTGCCACAGTGTCATATCCAA-3′); U6 (F: 5′-CTCGCTTCGGCAGCACA-3′, R: 5′-AACGCTTCACGAATTTGCGT-3′); GADPH (F: 5-′GAGAAGTATGACAACAGCCTC-3′, R: 5′-ATGGACTGTGGTCATGAGTC-3′). U6 or GAPDH was used as an internal control to normalize the relative gene expression using the 2^−ΔΔCq^ method [23].

### Cell viability

The viability of treated INS-1 cells was assessed by CCK-8 assay. Briefly, treated cells in each well of 96 well-plates were added with CCK-8 solution (10%, v/v) for 2 h incubation. Finally, the absorbance of each well at 450 nm was measured with a microplate reader (Bio-Tek, USA) to calculate cell viability.

### Western blot

Total protein of tissues or cells was extracted and quantified by RIPA lysis buffer and BCA Protein Assay Kit (Sigma-Aldrich, MO, USA), respectively. The samples were subjected to 10% SDS-PAGE gel for separation, followed by blot on PVDF membrane. Following blocking with skimmed milk (5%) for 30 min, the membranes were reacted with primary antibodies overnight at 4 °C, followed by incubation with the corresponding second antibody for 1 h. The ECL^™^ Western Blotting Analysis System (Sigma-Aldrich, MO, USA) was applied for visualizing specific protein bands of the membranes. ImageJ software was used for the semi-quantitative analysis of protein bands. All primary antibodies used in this study were purchased from Abcam (MA, USA): TFR1 (#ab1086), GPX4 (#ab219592), USP22 (#ab195289), SIRT1 (#ab ab110304), and GAPDH (#ab8245).

### Glucose-stimulated insulin secretion (GSIS)

GSIS was performed as previously reported [24]. In Brief, treated INS-1 cells were washed and starved in Krebs–Ringer bicarbonate (KRB) buffer for 90 min. Then, cells were incubated in KRB buffer containing varying glucose concentrations (basal, 2 mM; stimulatory, 25 mM). After additional 90 min incubation, the supernatant was harvested to analyze insulin content using the RIA method.

### Dual-luciferase reporter (DLR) assay

The putative binding sites for miR-144-3p in the 3′-UTR of USP22 mRNA were predicted by TargetScan [25]. DLR assay was performed to confirm their direct interaction. In brief, the sequence corresponding to the 3′-UTR of USP22 mRNA containing the wild-type (WT) or mutated (MUT) miR-144-3p binding sequence was inserted into a pmirGLO vector (Promega, WI, USA) to construct luciferase reporters. Then, USP22 WT or USP22 MUT luciferase reporter was co-transfected with miR-144-3p mimic or NC mimic into cells. Luciferase activity was measured with the Dual-Luciferase Assay (Promega, WI, USA) after 48 h transfection.

### Co-immunoprecipitation (Co-IP) assay

The total protein of HEK 293T cells with different transfections was extracted in Co-IP lysis buffer (Beyotime, Beijing, China). For immunoprecipitation, the cell lysate was incubated with anti-flag (#ab1162, Abcam) or anti-HA (#ab18181, Abcam) or anti-Myc (#ab32) antibody and Dynabeads Protein G at 4 °C overnight. Then, the beads were washed three times with lysis buffer, and the immunoprecipitates were harvested for further western blot analysis.

### In vitro ubiquitination assay

The transfected cells were harvested and lysed after incubated with 10 µM MG132 for 6 h. The ubiquitinated proteins were pulled down with anti-HA antibody following the manufacturer’s instructions. The protein complexes were then immunoblotted with anti-ubiquitin antibody (#ab140601; Abcam) to visualize the level of ubiquitination.

### Cycloheximide (CHX) assay

To detect protein stability, the transfected cells were mixed with 50 mg/mL CHX (an inhibitor of protein synthesis) and incubated for 0, 2, 4, 8 h, respectively. Then, the post-incubation cells were harvested and lysed, and the lysates were finally separated by SDS-PAGE for WB analysis to determine protein levels at each time point.

### Statistical analysis

All data was expressed as mean ± SD, and analyzed by GraphPad Prism 8.0 (CA, USA). Two-tailed Student’s t-test, Pearson’s correlation analysis and one-way analysis of variance (one-way ANOVA), were used as appropriate. Discrepancy was considered statistically significant if P < 0.05.

## Results

### Ferroptosis was observed in the pancreas of the T2DM mouse model

**Fig. 1:**
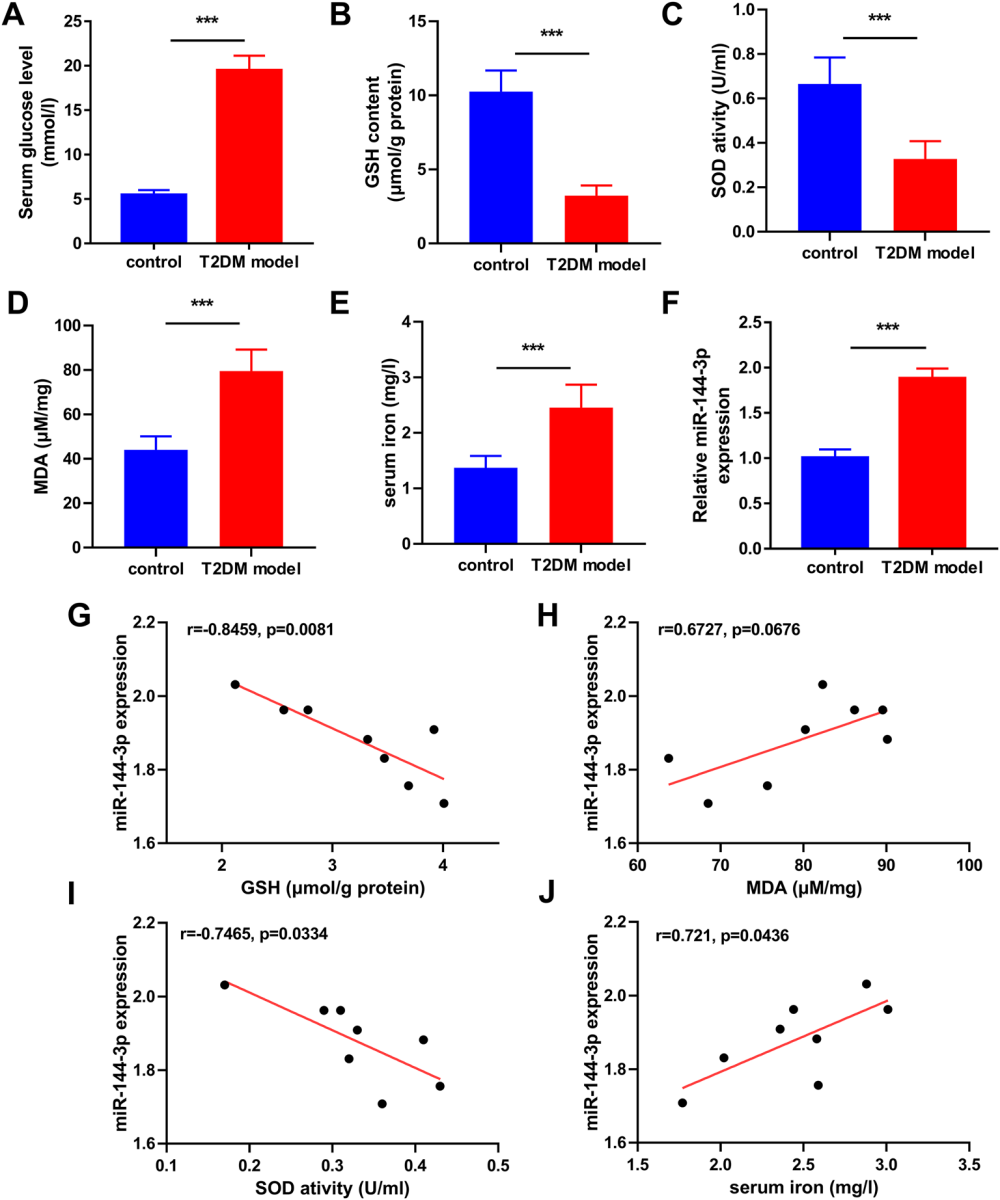
Evaluation of ferroptosis in the pancreas of T2DM mouse model. Pancreatic tissues were collected from T2DM mouse models (n = 8) as well as from normal mice (n = 8). **A** Serum glucose levels, **B** pancreatic GSH content, **C** pancreatic SOD activity, **D** pancreatic MDA levels, and **E** serum iron content were measured using the corresponding detection kits. **F** The expression of miR-144-3p in pancreatic tissues between the control and T2DM model groups. The correlations of miR-144-3p expression with **G** GSH content, **H** MDA levels, **I** SOD activity, and **J** serum iron content were evaluated using Person correlation coefficient (^***^P < 0.005)

The serum glucose levels of mice in the T2DM model group was significantly higher than those of the control mice (19.46 ± 1.667 vs. 5.449 ± 0.554 mM), which confirmed the successful establishment of the T2DM model (Fig. 1A). As known, ferroptosis is caused by excessive iron-dependent lipid peroxidation. Our study indicated that both GSH content and SOD activity of the pancreatic tissues were significantly reduced in T2DM models compared with the controls (Fig. 1B, C). In the meantime, the MDA level was conversely elevated in the pancreatic tissues of T2DM models (Fig. 1D). Moreover, the serum iron level of T2DM models was significantly higher than that in the controls (Fig. 1E). Considering that is blood glucose is closely related to the severity of T2DM, the correlation of blood glucose with GSH content, MDA content, SOD activity, and serum iron concentration were subsequently analyzed. The results revealed that the blood glucose of the T2DM models was positively correlated with GSH content, but inversely correlated with MDA content (Additional file 1: Fig. S1A, B). However, the correlation between the blood glucose of the T2DM models with SOD activity and iron concentration was not significant (Additional file 1: Fig. S1C, D). These data indicated that ferroptosis occurs in T2DM. Considering that miR-144-3p has been reported to be implicated in the pathogenesis of T2DM, we wondered whether miR-144-3p contributes to T2DM development by regulating ferroptosis and subsequently analyzed the correlation of miR-144-3p with GSH content, MDA content, SOD activity, and total iron concentration. Consistent with previous studies, miR-144-3p is highly expressed in the T2DM model (Fig. 1F). Interestingly, miR-144-3p expression of the T2DM models was positively correlated with serum iron level (Fig. 1J), but inversely correlated with MDA content and SOD activity (Fig. 1H, I). However, the correlation between the miR-144-3p expression of the T2DM models with GSH content was not significant (Fig. 1G). These results suggested that miR-144-3p might be involved in the ferroptosis in T2DM.

### Inhibition of miR-144-3p rescues high glucose-induced ferroptosis of INS-1 cell

**Fig. 2:**
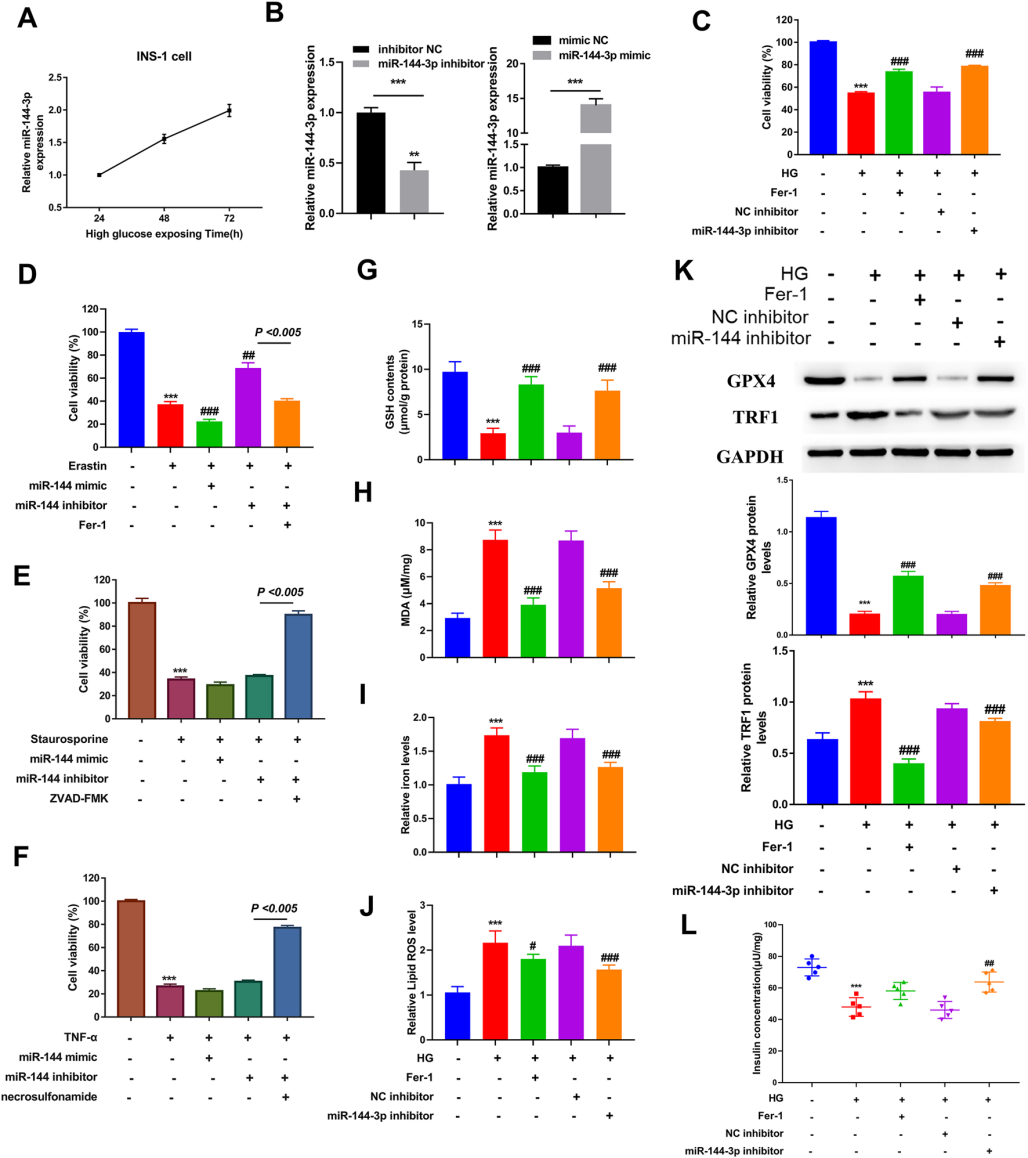
The effect of miR-144-3p on ferroptosis of INS-1 cells. The in vitro T2DM model was established by culturing INS-1 cells under high glucose (HG) condition. **A** The expression level of miR-144-3p in INS-1 cells after 24, 48, 72 h cultivation of HG was detected by RT-qPCR. **B** RT-qPCR detected the transfection efficiency of miR-144-3p inhibitor (left) and miR-144-3p mimic (right). **C** Under HG condition, the cell viability of INS-1 cells treating with or without miR-144-3p inhibitors or NC inhibitors; cell culturing under normal condition considered as negative control. **D** The cell viability of INS-1 cells treating with miR-144-3p mimics/inhibitors, erastin and Fer-1 (ferroptosis inducer and inhibitor) was detected by CCK-8 assay. **E** The cell viability of INS-1 cells treating with miR-144-3p mimics/inhibitors, staurosporine and ZVAD-FMK (apoptosis inducer and inhibitor). **F** The cell viability of INS-1 cells treating with miR-144-3p mimics, TNF-α and necrosulfonamide (necroptosis inducer and inhibitor). Under HG condition, INS-1 cells treating with Fer-1 or miR-144-3p mimics. **G** GSH content, **H** MDA levels, and **I** iron content of cells were measured using the corresponding detection kits. **J** Lipid ROS level was determined by the BODIPY™ 581/591 C11 method. **K** The levels of ferroptosis related proteins (GPX4 and TFR1) were evaluated by western blot. **L** Glucose stimulated insulin secretion was detected to assess β cells’ function (^***^P < 0.005 vs. INS-1 cells; ^##^P < 0.01 and ^###^P < 0.005 vs. HG-induced INS-1 cells)

In order to understand the role of miR-144-3p on the ferroptosis of pancreatic β cells, we used high glucose (HG) to stimulate INS-1 cells ferroptosis. The result revealed that the expression levels of miR-144-3p in INS-1 cell was increased with the cultivation time of HG (Fig. 2A). RT-qPCR analysis confirmed miR-144-3p inhibitor/mimic was successfully transfected into INS-1 cells (Fig. 2B). After treating with HG for 24 h, the cell viability of INS-1 cells was obviously impaired compared with the controls (Fig. 2C). Notably, ferrostatin-1 (Fer-1) could effectively counter HG-mediated impairing effect on INS-1 cells viability, suggesting ferroptosis has occurred in HG-induced INS-1 cells. Besides, silencing the expression of miR-144-3p exhibited a similar effect to the Fer-1 on HG-induced INS-1 cells (Fig. 2C). This result hinted that miR-144-3p is related to the HG-induced death in INS-1 cells. To determine which types of cell death is miR-144-3p involved in, we treated INS-1 cells with ferroptosis activator (erastin) and inhibitor (Fer-1), apoptosis activator (stauroporine) and inhibitor (ZVADFMK), and necrosis activator (TNF-α) and inhibitor (necrosulfonamid), respectively. Erastin, stauroporine, and TNF-α result in a significant reduction of cell viability in INS-1 cells (Fig. 2D–F). It was found that miR-144-3p knockdown effectively suppressed erastin-induced ferroptosis (Fig. 2D). However, miR-144-3p knockdown failed to rescue the apoptosis and necrosis of INS-1 cells (Fig. 2E, F). Thus, miR-144-3p is involved in ferroptosis to regulate INS-1 cells viability.

In agreement with the results of in vivo in T2DM model mice, HG caused a decrease in GSH content in INS-1 cells, as well as an elevation in MDA content, iron concentration, and lipid ROS level (Fig. 2G–J). As expected, all these roles could be significantly rescued by the Fer-1. On the other hand, silencing miR-144-3p also partly blocked the effect of HG on GSH, MDA, iron, and lipid ROS content in INS-1 cells (Fig. 2G–J). In addition, the expression levels of proteins related to ferroptosis, such as GPX4 and TFR1, were also detected in INS-1 cells with different treatments (Fig. 2K). We found that GPX4 protein expression was reduced in HG-induced INS-1 cells, whereas the expression of TFR1 protein exhibited an elevation. Both the treatment of Fer-1 and the transfection of miR-144-3p inhibitor reversed the effects of HG on ferroptosis-related proteins in INS-1 cells. Furthermore, we tested the insulin secretion ability of INS-1 cells by evaluating the pancreatic β cells’ function to explore whether ferroptosis affect the responsiveness of INS-1 cells to HG. Our results showed that HG significantly decreased the insulin secretion of INS-1 cells; and this deleterious role could be recused by Fer-1 intervention or the transfection of miR-144-3p inhibitor (Fig. 2L).

### USP22 is a direct target of miR-144-3p in the ferroptosis of INS-1 cells

**Fig. 3:**
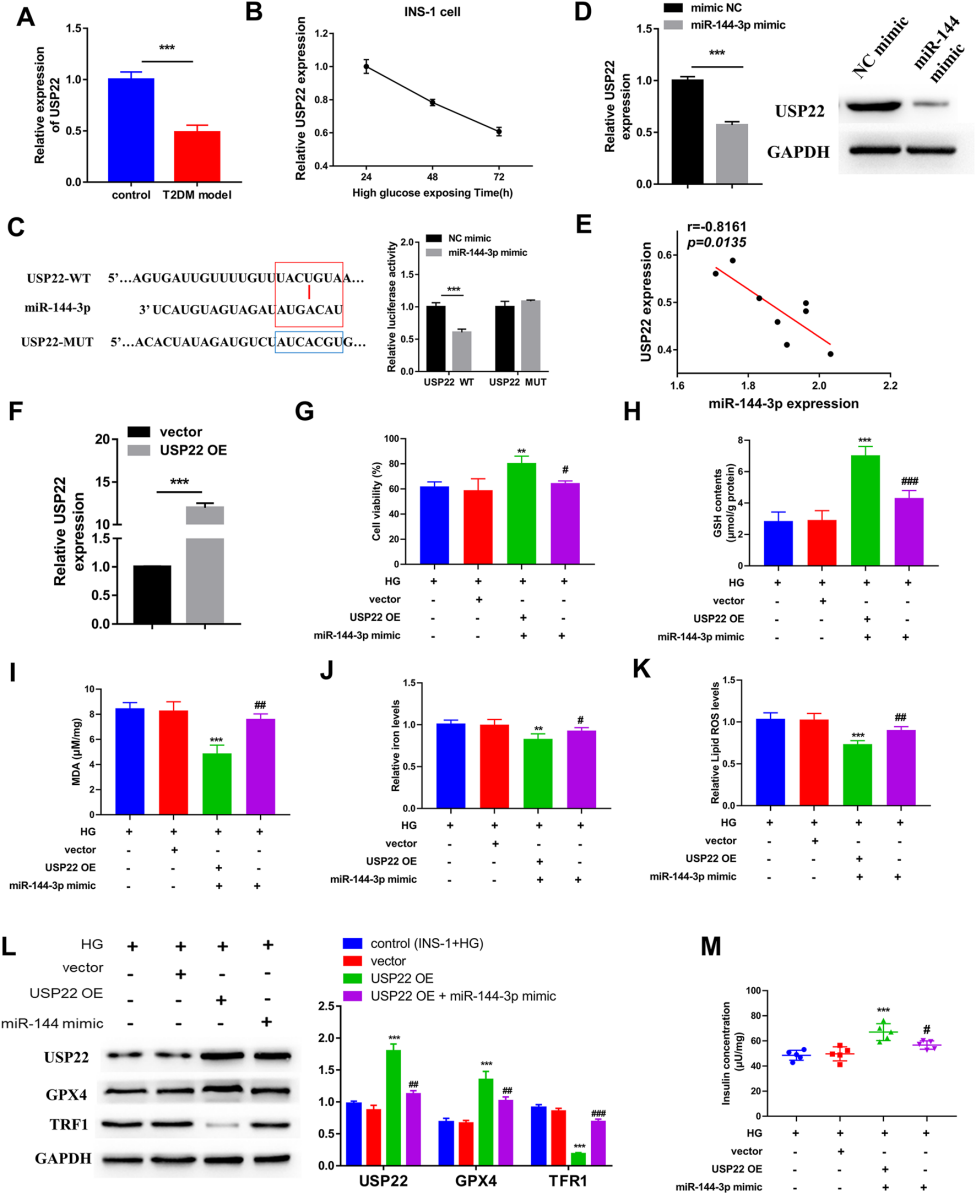
USP22 is a target of miR-144-3p and exerts a suppressive function on the ferroptosis of pancreatic β cells. **A** The expression of USP22 in pancreatic tissues between the control and T2DM model groups. **B** The expression level of USP22 in INS-1 cells after 24, 48, 72 h cultivation of HG was detected by RT-qPCR. **C** Dual-luciferase reporter assay confirmed the putative binding site for miR-144-3p in the 3′-UTR of USP22 (right), as predicted by the TargetScan (left). **D** The expression of USP22 in INS-1 cells transfected with miR-144-3p mimics at mRNA (left) and protein (right) levels. E The correlation analysis between the expression of miR-144-3p and USP22 in the pancreas of the T2DM model. **F** RT-qPCR detected the transfection efficiency of USP22 overexpression vector (USP22 OE). Before treating with HG, INS-1 cells transfected with or without USP22 OE alone, or together with miR-144-3p mimic or NC mimic. **G** Cell viability of HG-induced INS-1 cells was detected by CCK-8 assay. **H** GSH content, **I** MDA levels, and **J** iron content were measured using the corresponding detection kits. **K** Lipid ROS level was determined by the BODIPY™ 581/591 C11 method. **L** The levels of USP22 and ferroptosis-related proteins (GPX4 and TFR1) were evaluated by western blot. **M** Glucose stimulated insulin secretion was detected to assess β cells’ function (^**^P < 0.01 and ^***^P < 0.005 vs. HG-induced INS-1 cells; ^#^P < 0.05, ^##^P < 0.01, ^###^P < 0.005 vs. HG-induced INS-1 cells + USP22 OE)

It is widely accepted that the regulatory role of miRNAs in diverse diseases is attributed to their capacity of binding to the 3′UTR region of the target gene to suppress the expression of its target genes at the post‑transcriptional level [26]. Hence, we further explore the target gene of miR-144-3p in regulating ferroptosis. Based on online bioinformatics analysis, we found there is a presumptive binding site for miR-144-3p in the 3′-UTR of USP22, a ubiquitin-specific peptidase that has been reported to be down-regulated in HG treated INS-1 cells [27]. It was observed that the expression of USP22 in the control mice was significantly higher than that in the T2DM model mice (Fig. 3A). In addition, the expression levels of USP22 in INS-1 cell was reduced with the cultivation time of HG (Fig. 3B). A previous study demonstrated that USP22 has the potential of inhibiting ferroptosis [28]. Therefore, we hypothesized that miR-144-3p aggravates the ferroptosis of pancreatic β cells by targeting USP22, thereby promoting the progression of T2DM. DLR assay confirmed that miR-144-3p directly targeted and regulated USP22 expression (Fig. 3C). Then, both RT-qPCR and western blot analyses showed that the expression of USP22 in INS-1 cells could be down-regulated by the transfection of miR-144-3p mimics (Fig. 3D). We subsequently analyzed the correlation between miR-144-3p and USP22 expressions in both the pancreas of T2DM model mice, the result hinted there is an inverse correlation between the expressions of miR-144-3p and USP22 (Fig. 3E).

**Fig. 4:**
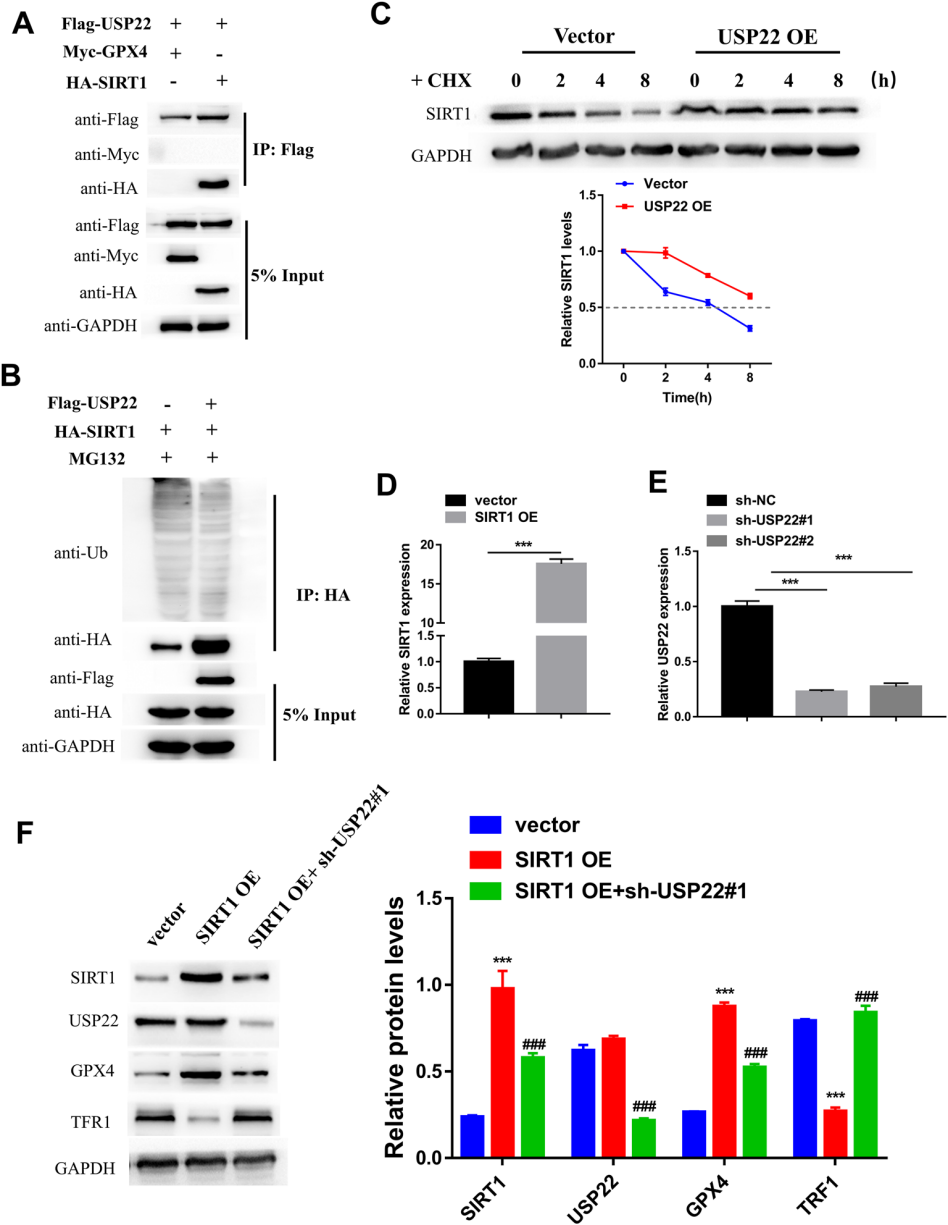
USP22 suppressed HG-induced ferroptosis in pancreatic β cells by stabilizing SIRT1 expression. **A** Co-IP was performed in cells transfecting with Flag-tagged USP22 and Myc-tagged GPX4 or HA-tagged SIRT1. Cell lysates were immunoprecipitated with anti-Flag antibodies. **B** In vitro ubiquitination assay was performed in cells transfecting with HA-tagged SIRT1 alone or together with Flag-tagged USP22; After transfection 48 h, cells were treated with MG132 for 6 h. Cell lysates were subjected to immunoprecipitated with anti-HA antibodies. **C** Cells were transfected with USP22 OE and harvested at 0, 2, 4, and 8 h after treatment with CHX to detect the expression of SIRT1 by western blot. RT-qPCR detected the transfection efficiency of **D** SIRT1 overexpression vector (SIRT1 OE) and **E** USP22 short hairpin RNA (sh-USP22#1 and sh-USP22#2). **F** The levels of SIRT1, USP22, and ferroptosis-related proteins (GPX4 and TFR1) were evaluated by western blot (^***^P < 0.005 vs. vector; ^###^P < 0.005 vs. SIRT1 OE)

In order to explore the functional relevance of USP22/miR-144-3p interaction on ferroptosis of pancreatic β cells, we transfected USP22 OE alone or together with miR-144-3p mimic into INS-1 cells. RT-qPCR showed that the transfection efficiency of USP22 OE is excellent (Fig. 3F). CCK-8 assay showed that overexpressing USP22 in INS-1 cells could rescue the cell viability suppression induced by HG (Fig. 3G). On the other hand, the effect of USP22 OE on the cell viability of HG-induced INS-1 cells was partly reversed by miR-144-3p mimic (Fig. 3G). In the context of HG-induced ferroptosis, USP22 OE led to a significant increase in GSH content and iron level in INS-1 cells, as well as a reduction in MDA content and lipid ROS level; but these effects of USP22 OE could be counteracted in response to miR-144-3p mimic (Fig. 3H–K). Unsurprisingly, the expression level of USP22 protein in HG-induced INS-1 cells was dramatically increased after transfecting USP22 OE. This increase was blocked in part by miR-144-3p mimic (Fig. 3L). Likewise, the increase of GPX4 protein level, as well as the decrease of TFR1 protein level that caused by USP22 OE in HG-induced INS-1 cells were also partly abrogated by miR-144-3p mimic (Fig. 3L). The result of GSIS assay further indicated that overexpressing USP22 could rescue the insulin secretion ability of INS-1 cells that was impaired by HG; but miR-144-3p mimic abated this impact (Fig. 4H).

### USP22 suppressed HG-induced ferroptosis in pancreatic β cells by stabilizing SIRT1 expression

Then, the molecular mechanism underlying the effect of USP22 on HG-induced ferroptosis in pancreatic β cells was further illustrated. Since the USP22 is a ubiquitin-specific peptidase that can regulate its target protein expression via deubiquitination function, Co-IP and in vitro ubiquitination assay was performed to investigate whether USP22 can bind to and stabilize the negative regulator protein of ferroptosis for suppressing ferroptosis. Co-IP results showed that USP22 interacted with SIRT1, but not GPX4 (Fig. 4A). The analysis on the ubiquitination level of SIRT1 showed that the SIRT1 ubiquitination level reduced significantly after overexpressing USP22 in INS-1 cells (Fig. 4B), demonstrating that USP22 weakens the ubiquitination level of SIRT1. Furthermore, CHX assay was also performed to explore whether USP22 affected SIRT1 stability. Our data showed that USP22 overexpression increased the stability of SIRT1, thereby prolonging the half-life of SIRT1 protein (Fig. 4C). Furthermore, we also investigated the effect of USP22/SIRT1 on ferroptosis-related protein expression by transfecting SIRT1 OE alone or together with sh-USP22 into INS-1 cells. The transfection of SIRT1 OE or sh-USP22 successfully overexpressed SIRT1 or silenced USP22 in INS-1 cells (Fig. 4D, E). Western blot analysis showed that overexpressing SIRT1 led to a significant increase in GPX4 expression and a reduction in TFR1 expression, but there was no influence in USP22 expression (Fig. 4F). After silencing USP22 in SIRT1 overexpressing cell, the expression of SIRT1 was significantly decreased; simultaneously, the effect of SIRT1 overexpression on GPX4 and SIRT1 expression was also partly reversed (Fig. 4F). Taken together, our results indicated that USP22 participated in proteasome degradation of SIRT1 to regulate the ferroptosis in pancreatic β cells.

### miR-144-3p antagomir has a potential anti-hyperglycemic effect in T2DM mice by attenuating ferroptosis

**Fig. 5:**
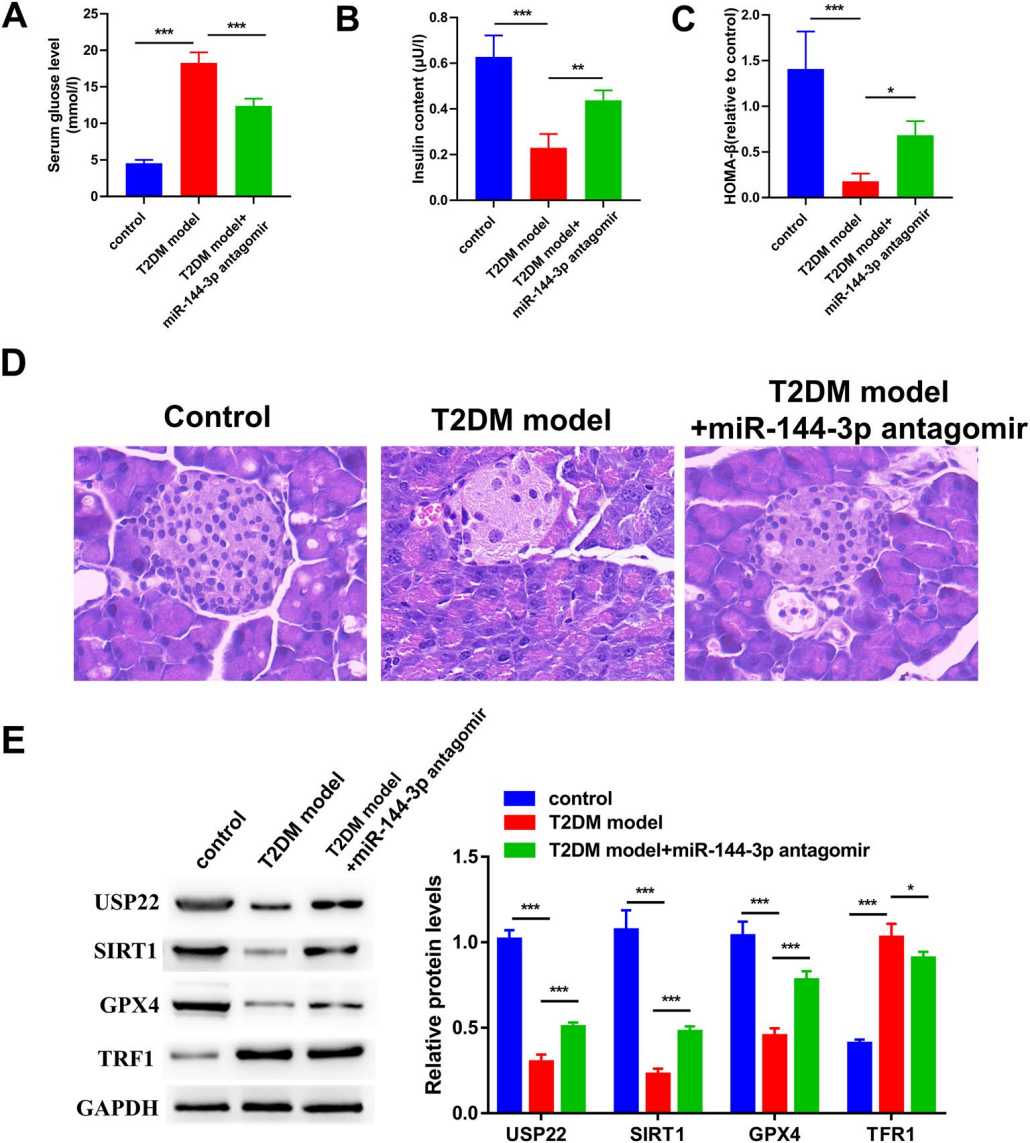
Anti-hyperglycemic effect of miR-144-3p antagomir in the T2DM mouse model. The serum **A** glucose and **B** insulin levels were detected using the corresponding detection kits. **C** The homeostasis model assessment of pancreatic β-cells, (HOMA-β) = 20 × fasting insulin/ (fasting blood glucose-3.5). **D** Representative images of hematoxylin and eosin (H&E) staining for the pancreas from diverse groups. **E** The levels of SIRT1, USP22, and ferroptosis-related proteins (GPX4 and TFR1) were evaluated by western blot (^*^P < 0.05, ^**^P < 0.01, and ^***^P < 0.005)

We further explored whether the inhibition of miR-144-3p expression conferred any anti-hyperglycemic effects in the T2DM mice. Compared with the control group, T2DM mice showed increased serum glucose level (Fig. 5A) and decreased insulin content (Fig. 5B). miR-144-3p antagomir partly prevented the increase of serum glucose level and the reduction of insulin content in T2DM mice. After calculating the HOMA-β index, we found that the HOMA-β of T2DM mice was obviously lower than that of the controls, and the treatment of miR-144-3p antagomir could partly elevate the HOMA-β of T2DM mice (Fig. 5C). H&E staining of pancreatic sections from T2DM model group displayed a substantial depletion in islets of Langerhans (Fig. 5D). Administration of miR-144-3p antagomir resulted in a marked enhancement in the islets’ size and the number of cells. We further detected the expression levels of ferroptosis-related proteins in the pancreas (Fig. 5D). The results revealed that the reduction in SIRT1, USP22, GPX4 protein levels, as well as the increase in TFR1 protein level, were both attenuated upon treatment with miR-144-3p antagomir (Fig. 5E).

## Discussion

It is widely accepted that pancreatic β cells death and dysfunction is a pivot of the strikingly promoted T2DM progression. Ferroptosis is a novel non-apoptotic regulated cell death form, which is characterized by the accumulation of lipid peroxidation products in an iron-dependent manner. Recent studies implicated ferroptosis in the pancreatic β cells of T2DM [29, 30]. However, the molecular mechanisms underlying ferroptosis that occurred in pancreatic β cells are largely undefined. Our study identified that ferroptosis is involved in the pathogenesis of T2DM. Abnormal expression of miR-144-3p is involved in the ferroptosis in INS-1 cells under HG conditions. Suppressing the expression of miR-144-3p could not only improve the viability of HG-induced INS-1 cells, but also attenuate the hyperglycemic symptom of T2DM mouse models. Additionally, we also preliminarily explored the mechanisms underlying the effect of miR-144-3p on ferroptosis of pancreatic β cells.

Initially, we detected and compared the GSH content, SOD activity, MDA, and iron levels in the pancreas from the control and T2DM mice. These indexes are widely used to evaluate the degree of lipid peroxidation, which could help to judge whether ferroptosis occurs [31]. HG could cause overload of iron that predisposes to oxidative stress in pancreatic islet, which leads to ferroptosis finally [32]. The antioxidant defense of pancreatic islet is weaker than other tissues, making it susceptible to the regulatory abnormality of ROS generation [33]. In this study, GSH content and SOD activity were reduced, but the MDA and serum iron levels were elevated in the T2DM mice compared with the controls. Moreover, it was observed that the serum glucose level was significantly correlated GSH content and MDA level in T2DM mice. These data demonstrated that ferroptosis likely occurs in the pancreas of T2DM mice. Several studies identified miR-144-3p as a biomarker of T2DM, of which aberrant up-regulation was involved in T2DM progression by impairing insulin signaling and promoting adipogenesis [18, 34]. Li et al. [35] recently demonstrated that miR-144-3p participates in modulating the intracellular level of both GSH and MDA. Interestingly, our data showed the expression level of miR-144-3p was positively correlated with GSH content and SOD activity, but negatively correlated with serum iron level in the T2DM mice. Hence, we speculated that miR-144-3p is involved in the regulation of ferroptosis in T2DM.

Previous studies documented that HG could reduce INS-1 cell viability and functions by inducing ferroptosis [29, 30]. Then, we stimulated INS-1 cells with HG in order to induce ferroptosis in vitro. Since there is no direct approach for detecting ferroptosis at present, most studies tend to add ferroptosis inhibitors including Fer-1, and subsequently detect the change in cell viability, to indirectly investigate ferroptosis [36, 37]. Our study showed that the treatment of Fer-1 blocked the reduction in cell viability induced by HG in INS-1 cells, verifying that ferroptosis does exist in HG-induced INS-1 cell death. Ferroptosis occurs as a result of the accumulation of lipid ROS levels [38]. So, we also detected the lipid ROS level of INS-1 cell to investigate the ferroptosis. Previous studies showed that HG stimulation led to the impaired cell viability and the accumulation of ROS, lipid peroxides and iron in β cells [29, 39]. Our study observed the similar result, and found that silencing miR-144-3p could rescue the impaired cell viability of INS-1 cells owing to HG. Simultaneously, the suppression of miR-144-3p caused an increase in the GSH content and lipid ROS levels in HG-induced INS-1 cells, as well as a decrease in the MDA and iron concentrations. These data demonstrated that the suppression role of miR-144-3p in the cell viability of HG-induced INS-1 is likely related to its promotion effect on ferroptosis. Moreover, USP22 is identified as a direct target gene of miR-144-3p, of which overexpression also increased the cell viability of HG-induced INS-1 cells. The detection of ferroptosis-related proteins also confirmed that HG-induced ferroptosis in INS-1 cells. As an important marker of ferroptosis, GPX4 can transform lipid hydroperoxides into lipid alcohols to attenuate ROS-mediated lipid peroxidation by using GSH, thereby protecting cells from ferroptosis [40, 41]. Li et al*.* demonstrated that quercetin restrained ferroptosis of pancreatic β cells by up-regulating GPX4 29. Our data revealed that miR-144-3p contributes to HG-induced ferroptosis by directly suppressing USP22 expression, which turns to change the expression of GPX4 and TFR1, ultimately impairing the cell viability and insulin secretion function of β cells. Cystine, a critical precursor for GSH synthesis, is mainly transported from the extracellular to intracellular by the xC^−^ system, which is modulated by SLC7A11 expression [42]. It has also been reported that HG is capable of promoting the activation of p53 [43]. A previous study revealed that p53 could sensitize cells to ferroptosis via down-regulating SLC7A11 [44]. A recent study uncovered that USP22 can repress the transcriptional activity of p53 to upregulate SLC7A11 expression, thereby suppressing ferroptosis in cardiomyocytes [28]. The authors revealed that these effects of USP22 relied on its deubiquitination function for SIRT1. Similarly, our study also identified SIRT1 as a downstream molecule of USP22, and stabilization of SIRT1 by USP22 caused GPX4 upregulation and TRF1 downregulation in INS-1 cells. SIRT1 has been reported to confer widespread influence on several processes, and its dysregulation is closely related to the development of many diseases, including T2DM [45]. This suggested SIRT1 might be a mediator of miR-144-3p/USP22 in regulating the ferroptosis of INS-1 cells. Finally, in vivo study proved that miR-144-3p antagomir could restore the impaired function of pancreatic β-cells by suppressing ferroptosis to exert anti-hyperglycemic effect on T2DM mice.

**Fig. 6:**
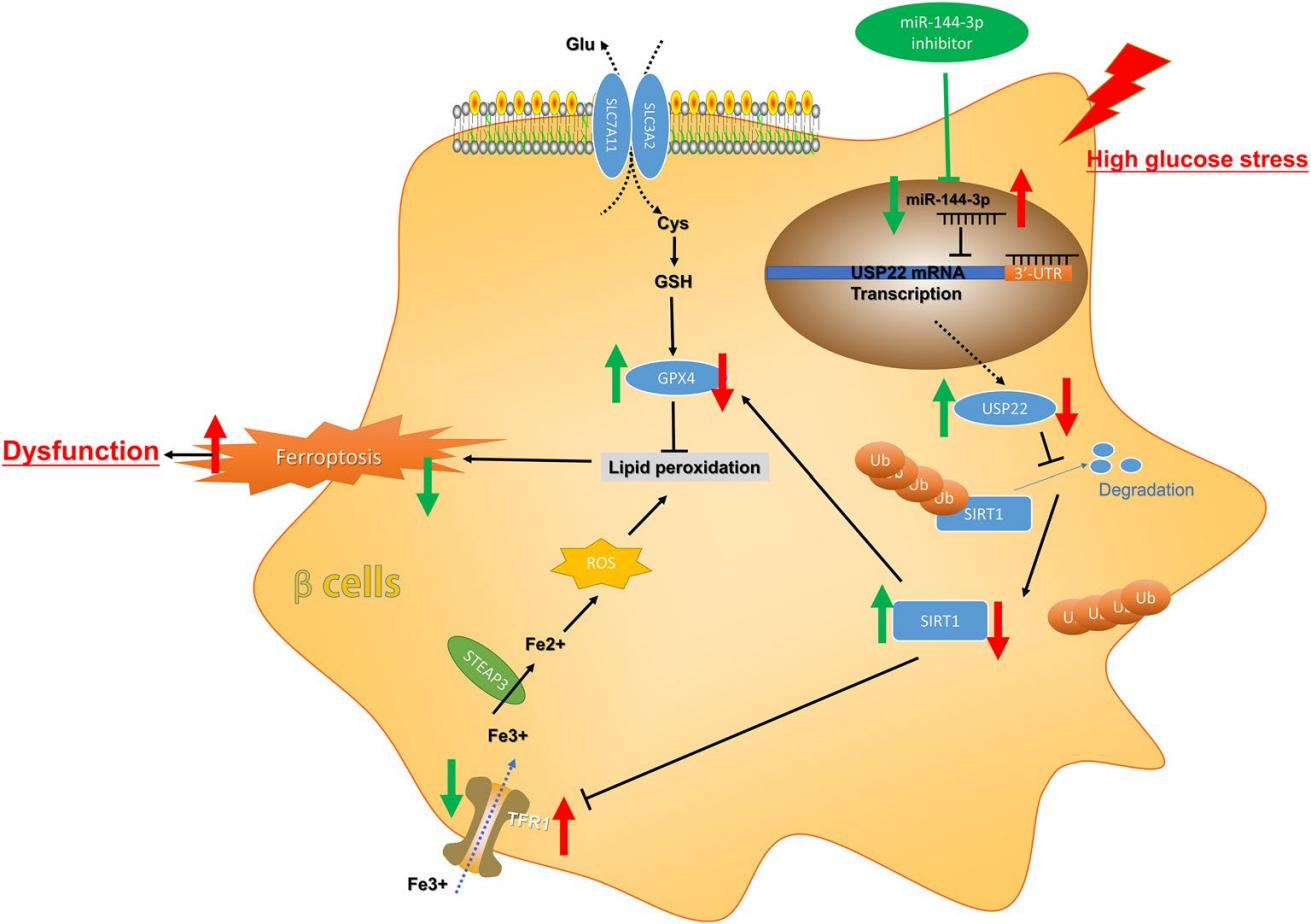
Graphically summarized finding of this study

In short, the abnormal up-regulation of miR-144-3p clearly suppressed the expression of USP22 mRNA and inhibited its downstream effects on the ferroptosis pathway to cause the dysfunction of pancreatic β cells (Fig. 6), thereby promoting the progression of T2DM. Hence, miR-144-3p/USP22 interaction might be a crucial event that regulated ferroptosis during T2DM development, which served as a novel target for T2DM treatment. It should be acknowledged that, however, the present study is preliminary nature. Further studies are required to perform at the clinical and mechanistic levels in future.

## Conclusion

In conclusion, aberrantly up-regulated miR-144-3p triggered the ferroptosis in pancreatic β cells in the context of HG through the down-regulation of USP22/SIRT1. Suppression of miR-144-3p attenuated hyperglycemic symptoms and the ferroptosis of pancreas in the T2DM mouse model, providing a theoretical foundation for the future development of novel strategies for T2DM treatment.

## Supplementary Information

**Additional file 1: Figure S1.** The correlations of serum glucose level with **(A)** GSH content, **(B)** MDA levels, **(C)** SOD activity, and **(D)** iron content were evaluated using Person correlation coefficient.

## Data Availability

The datasets used and/or analysed during the current study are available from the corresponding author on reasonable request.

## Abbreviations

T2DM: Type 2 diabetes mellitus
HG: High glucose
GPX4: Glutathione peroxidase 4
GSH: Glutathione
xC-: Cysteine-glutamate transporter
miRNAs: MicroRNAs
ROS: Reactive oxygen species
HFD: High-fat diet
STZ: Streptozotocin
H&E: Hematoxylin–eosin
RIA: Radioimmunoassay
SOD: Superoxide dismutase
MDA: Malondialdehyde
HOMA: Homeostasis model assessment
GSIS: Glucose-stimulated insulin secretion
KRB: Krebs–Ringer bicarbonate
DLR: Dual-luciferase reporter
WT: Wild-type
MUT: Mutated
Co-IP: Co-immunoprecipitation
CHX: Cycloheximide
Fer-1: Ferrostatin-1
