# Supplementary figures and images for: Targeting ferroptosis with miR-144-3p to attenuate pancreatic β cells dysfunction via regulating USP22/SIRT1 in type 2 diabetes
Source: Diabetol Metab Syndr. 2022 Jun 27;14:89. doi: 10.1186/s13098-022-00852-7 (PMC9235078; doi:10.1186/s13098-022-00852-7)

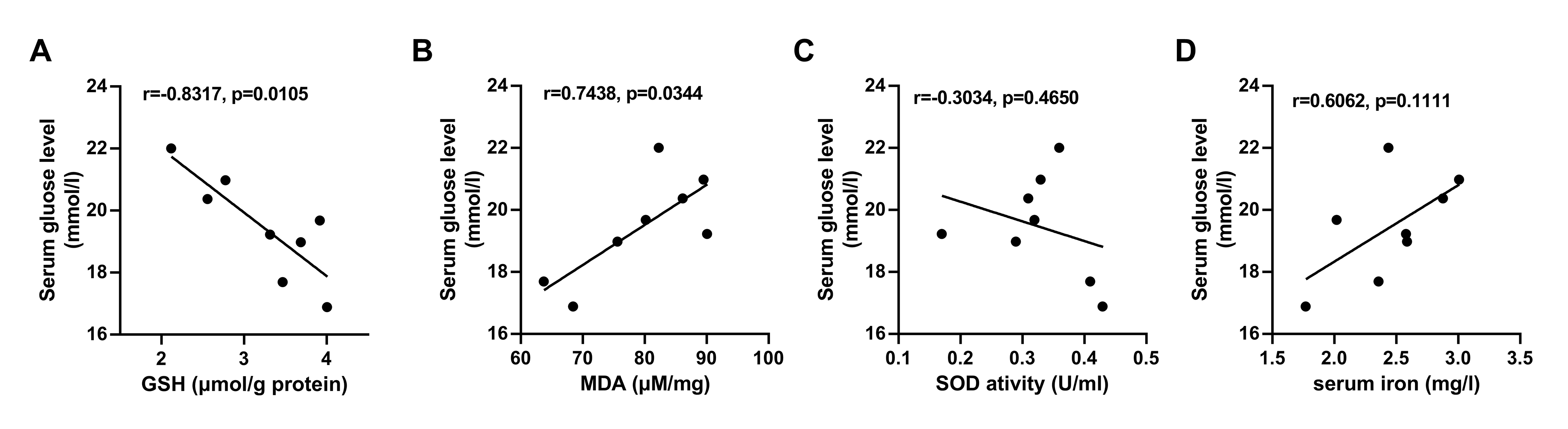

Supplement: Supplementary file 1 — Additional file 1: Figure S1. The correlations of serum glucose level with (A) GSH content, (B) MDA levels, (C) SOD activity, and (D) iron content were evaluated using Person correlation coefficient. [file 13098_2022_852_MOESM1_ESM.tif]
